# Bacteriotherapy with Streptococcus salivarius 24SMB and Streptococcus oralis 89a nasal spray for treatment of upper respiratory tract infections in children: a pilot study on short-term efficacy

**DOI:** 10.1186/s13052-020-0798-4

**Published:** 2020-04-03

**Authors:** Sara Manti, Giuseppe Fabio Parisi, Maria Papale, Amelia Licari, Carmelo Salpietro, Michele Miraglia del Giudice, Gian Luigi Marseglia, Salvatore Leonardi

**Affiliations:** 1 Department of Pediatrics, Unit of Pediatric Genetics and Immunology, University of Messina, Via Consolare Valeria 1, 98125 Messina, Italy; 2 Department of Clinical and Experimental Medicine, University of Catania, Via Santa Sofia 78, 95123 Catania, Italy; 3 Pediatric Clinic, Department of Pediatrics, Fondazione IRCCS Policlinico San Matteo, University of Pavia, Via Camillo Golgi 17, 27100 Pavia, Italy; 4 Department of Woman, Child and of General and Specialized Surgery, University of Campania “Luigi Vanvitelli.”, Via Luigi De Crecchio 2, 80138 Naples, Italy

**Keywords:** Bacteriotherapy, Children, RRIs, Treatment, URTI

## Abstract

**Background:**

Recurrent respiratory infections (RRIs) are defined by the presence of at least one of the following criteria: (i) > 6 annual respiratory infections (RIs); (ii) > 1 monthly RIs involving the upper airways from September to April; (iii) > 3 annual RIs involving the lower airways represent a very common health problem in the first years of life. We conducted a multi-centre, prospective, single-open study to assess the efficacy and the safety of *Streptococcus salivarius* 24SMBc and *Streptococcus oralis* 89a in the prevention of upper respiratory tract infections (URTIs) in children.

**Methods:**

Ninety-one children (M:F = 47:44, mean age 7.4 ± 2.3 years) with RRIs were enrolled in the study between September and November 2018. At baseline, children received *Streptococcus salivarius* 24SMBc and *Streptococcus oralis* 89a as 2 puffs for nostril twice/day for 7 days/months. The treatment lasted for 3 consecutive months. Efficacy was expressed in terms of absence or presence of fever, cough, bronchospasm, rhinorrhea and otalgia, at 1 month (T1), and 3 (T3) months. Safety and tolerability of the probiotic were evaluated on the basis of the number and type of adverse events (AEs) recorded during the treatment.

**Results:**

Children treated with *Streptococcus salivarius* 24SMBc and *Streptococcus oralis* 89a showed a significant decrease of symptoms including episodes of fever, cough, bronchospasm, rhinorrhea, and otalgia (*p* < 0.001) compared to baseline. The treatment significantly reduced the number of episodes of fever, cough, bronchospasm, rhinorrhea, otalgia, and cough also in patients with positive familial history for atopy and in atopic children (*p* < 0.05). No significant differences in symptoms among children with negative familial history for atopy and children with positive familial history for atopy subgroups, not atopic and atopic children subgroups, and smoke-exposed and not smoke-exposed subgroups were observed (*p* > 0.05). Conducting a subgroup analysis according to the age, it has been reported that children aged 1–3 years old showed an improvement in all symptoms, however, they become statistically significant only at the end of the 3 months of treatment (*p* < 0.05). Conversely, in children aged 3–6 and 6–12 years old, the therapeutic efficacy was progressive and significant already from the first month of therapy (*p* < 0.05). None of the children were withdrawn from the study because of AEs, although 9 children experienced burning nose leading to interruption of therapy.

**Conclusions:**

Our findings suggest that Streptococcus salivarius 24SMBc and Streptococcus oralis 89a treatment is safe and seems to be effective on short-term in the treatment of RRIs. Studies involving a longer observation period are necessary to establish the real efficacy of the product for the treatment of pediatric patients affected by RRIs.

## Background

Recurrent respiratory infections (RRIs) are characterized by significant morbidity and represent a very common health problem in the first years of life, requiring multiple physician visits and often hospitalisation, with significant implications for the patient’s family, the paediatrician and the pharma-economy [1].

At least one of the following criteria has to be present to diagnose RRIs: (i) > 6 annual respiratory infections (RIs); (ii) > 1 monthly RIs involving the upper airways from September to April; (iii) > 3 annual RIs involving the lower airways [1]. In accordance to the affected anatomic locations, the RIs are classified into upper respiratory tract infections (URTIs) - otitis, rhinitis, sinusitis, pharyngo-tonsillitis- and lower respiratory tract infections (LRTIs) - wheezing, bronchitis, bronchiolitis, and pneumonia [2, 3].

It has been estimated that approximately 6% of children suffer from RRIs. This reported incidence could be related to several factors such as genetic, immunological (e.g., anatomic and functional alteration in the respiratory tract, increased exposure to infectious agents, atopy, and immunodeficiency) [4], social and environmental (e.g., day-care attendance, physical stress, duration of breast-feeding, family size, air pollution, pets at home, parental smoking, missed vaccination) [5], anthropometric (e.g., age, sex, prematurity, low birth weight) factors and comorbidities (e.g., cardiopulmonary, gastrointestinal, neurological) [6–8].

According to the etiology, a treatment should be initiated [9], however, studies reported that RRIs can be often transient and resolve by itself, thus, any specific treatment is required [10]. On the other hand, the recurrence of the RIs leads to an increased risk of misdiagnosis and, consequently, to an unnecessary prescription antibiotic therapy, also, contributing to drug resistance development [11]. Recently, to prevent RRIs and avoid inappropriate therapy as well as to reduce the incidence of drug resistance, *alternative* treatments have been proposed [12]. An interesting way has been highlighted by the study of nasal microbiome that, interacting with the local epithelial and immune cells, evoking systemic immune responses and, eliminating the invading species, acts as a “health friendly bacteria” [13, 14]. In light of these beneficial properties, authors successfully looked to the vital bacteria, better known as probiotics, for reinforcement of microbiome homeostasis as an additional and effective weapon against RRIs [15–17]. With this regard, *Streptococcus salivarius* 24SMB and *Streptococcus oralis* 89a, belonging to α-hemolytic strains, have been reported to be safe and well tolerated when administered in healthy subjects as well as in patients with RRIs [18, 19].

However, despite to these encouraging findings, the clinical evidence on the role of the “bacteriotherapy” approach on RRIs treatment is still limited. Thus, in order to fill this gap, we conducted a preliminary, prospective, single-open, multi-centre study to determine the efficacy and the safety of *Streptococcus salivarius* 24SMBc and *Streptococcus oralis* 89a in the treatment of URTIs in children.

## Methods

### Study design

A prospective, single-open, multi-centre study protocol was designed.

### Efficacy, safety and tolerability

Efficacy was expressed in terms of absence or presence of symptoms, such as fever, cough, bronchospasm, rhinorrhea and otalgia, at 1 month (T1), and 3 (T3) months.

Safety and tolerability of the probiotic were evaluated on the basis of the number and type of adverse events (AEs) recorded during the treatment, and according to the principles of good clinical practice*.*

### Subjects and eligibility criteria

A prospective, single-open, multi-centre study protocol was designed.

One hundred caucasian children with RRIs (57 males and 43 females) who had been referred to the Department of Clinical and Experimental Medicine, University of Catania, to the Section of Immuno-Allergoloy, Department of Pediatrics, University of Messina, to Pediatric Clinic, Fondazione IRCCS Policlinico San Matteo, University of Pavia, and to Department of Woman, Child and of General and Specialized Surgery, University of Campania between September and November 2018, were enrolled in the study.

URTIs were diagnosed in 100 children. The symptom frequency (single and combined symptoms) was > 3 episodes in the preceding 6 months or > 4 episodes in the preceding 12 months from starting treatment, with a minimum interval of 10 days between episodes.

Inclusion criteria were: being out-patients of both sexes, aging 1–12 years, day care or primary school (children aged between 5 and 11 years), positive history for RIs featured by fever, cough, bronchospasm, persistent or recurrent rhinorrhea, and children with positive history otalgia.

The investigator collected the symptoms recorded by the parent in the study diary during the 3 months prior the start of treatment.

In accordance to their demographic and clinical characteristics, the enrolled population was divided into different subgroups: familial history of atopy-based groups (positive vs. negative); presence of allergy (atopic vs. not atopic groups); broad age-based groups (1–3, 3–6 and, 6–12 years old); and based groups smoking (exposed vs. unexposed).

Exclusion criteria included: associate diseases (hepatic, infectious, or endocrine diseases, genetic syndromes, immunodeficiencies, neurological and psychiatric problems), pulmonary or cardiac congenital malformations, chronic pulmonary disease (broncho-pulmonary dysplasia, cystic fibrosis, bronchiolitis obliterans post viral infection), severe atopic asthma, and congenital and/or acquired craniofacial anomalies. A washout period of 1 month for any treatments (e.g., immunomodulants, homeopathic therapy, or systemic corticosteroids, antiallergic drugs (i.e., nasal corticosteroids, antileukotrienes, cromones)), capable of interfering with the results was required. Patients with poor medication adherence were also excluded. Poor medication adherence was assessed by collecting detailed anamnestic data.

Institutional Review Board both of University of Catania and University of Messina approved the study. A written informed consent was obtained from the parents and informed assent from the children and adolescents.

### Study medication

*Streptococcus salivarius* 24SMBc and *Streptococcus oralis* 89a nasal spray was administered to enrolled pediatric population. The suspension consisted of a minimum of 10^9^ colony-forming units (CFU) per dose. This formulation was requiring refrigerator storage. The intranasal spray applicator was removed from the refrigerator approximately 30 min before administration to allow the solution to reach room temperature (not more than 25 °C), avoiding side effects such as itching or burning nose.

During the clinical trial, all drugs required to concomitant diseases were anyway prescribed, except for immunostimulants. Antibiotics, anti-inflammatory, and antipyretic drugs treatment could be administered if needed. As rescue medication for allergic children, an antihistamine (desloratadine) was allowed for symtomatic use alone.

### Study procedures

At the first visit, *Streptococcus salivarius* 24SMBc and *Streptococcus oralis* 89a were prescribed to 100 children in open manner. Ten days after discontinuing any treatment capable of interfering with the results as well as after a nasal saline lavage regimen *Streptococcus salivarius* 24SMBc and *Streptococcus oralis* 89a were administered as 2 puffs for nostril twice/day for 7 days/months. The treatment lasted for 3 consecutive months.

The children were examined at study entry, at 1 and 3 months (treatment period). To monitor the clinical course and assess treatment response, the parents of intervention children were telephonically contacted after 1 and 3 months and were asked a set of standard questions pertaining to the: 1) report of symptoms until the start of treatment; 2) report of symptoms during the 1st and the 3 month’s treatment; 3) safety and tolerability of the administered medication.

At T1 and T3, the investigator also summarized the data telephonically obtained and checked for the treatment adherence.

### Skin Prick Test and IgE measurement

Skin Prick Test (SPT) and IgE measurement were performed at baseline. Particularly, SPT was performed using a panel of aeroallergens (including: house dust mite, mixed grass, Parietaria, birch, olive tree, Alternaria, epithelium cat, and epithelium dog- Allergopharma, Reinbek, Germany). Normal saline and histamine were used as negative and positive controls, respectively. Skin wheal diameter was recorded at 15 min as the mean of 2 perpendicular measurements. A positive response was defined as a skin wheal diameter of 3 mm or more compared to negative control [2].

Total serum IgE levels were also determined in all subjects by ImmunoCAP100 system (Phadia, Uppsala, Sweden).

### Data analysis

The data collected were statistically analyzed by the statistical computer software SPSS, version 15.0. A *p* value less than 0.05 were considered statistically significant. Values were calculated as mean and standard deviation, χ^2^ test was used for comparisons between percentages. A *p*-value < 0.05 was considered significant.

## Results

Of all 100 enrolled subjects, only 91 completed the study and were considered in the final analysis. Demographic and baseline variables were reported in Table 1.

**Table 1:** Demographic and clinical findings of the enrolled pediatric population

	*RIs children
**N. evaluated children**	91
Age (years) (mean/SD*)	7.4 ± 2.3
*1–3 years old*	20
*3–6 years old*	42
*6–12 years old*	29
**Gender Male/Female**	47/44
**N. of children with family history of atopy/ without family history of atopy**	49/42
**N. of children with allergy/without allergy**	35/56
**N. of children exposed smoke/ unexposed smoke**	45/46

*RIs recurrent infections, *SD* standard deviation

### Efficacy assessment

Since the first month of treatment children treated with *Streptococcus salivarius* 24SMBc and *Streptococcus oralis* 89a showed a significant decrease in symptoms including episodes of fever, cough, bronchospasm, rhinorrhea, and otalgia (*p* < 0.001).

Specifically, a significant decrease in the number of the episodes of fever *p* < 0.001), bronchospasm (*p* < 0.001), rhinorrhea (*p* < 0.001), and otalgia (*p* < 0.001) has been observed at the first month of treatment. No further improvement was recorded at the third month of the treatment (fever *p* > 0.05), bronchospasm (*p* > 0.05), rhinorrhea (*p* > 0.05), and otalgia (*p* > 0.05)).

**Fig. 1:**
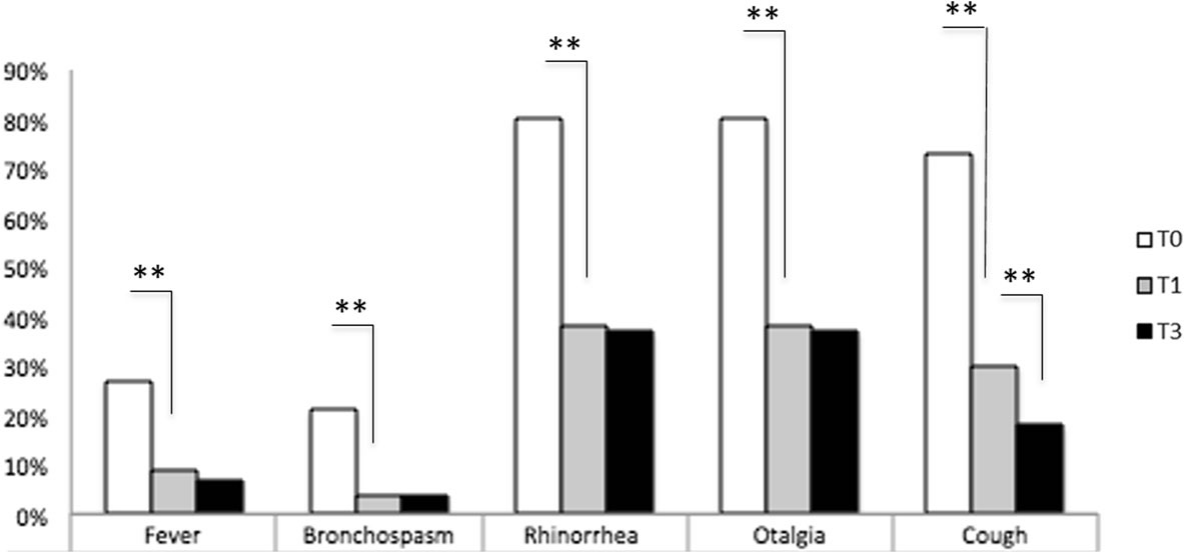
Efficacy assessment. *Streptococcus salivarius* 24SMBc and *Streptococcus or*alis 89a showed a significant decrease of symptoms including episodes of fever, cough, bronchospasm, rhinorrhea, and otalgia (**p < 0.001)

Conversely, a progressive and significant decrease in episodes of cough has been reported up to the third month of therapy (*p* < 0.001) (Fig. 1).

### Subgroup analysis

The efficacy of treatment with *Streptococcus salivarius* 24SMBc and *Streptococcus oralis* 89a was recorded since the first month of therapy.

**Fig. 2:**
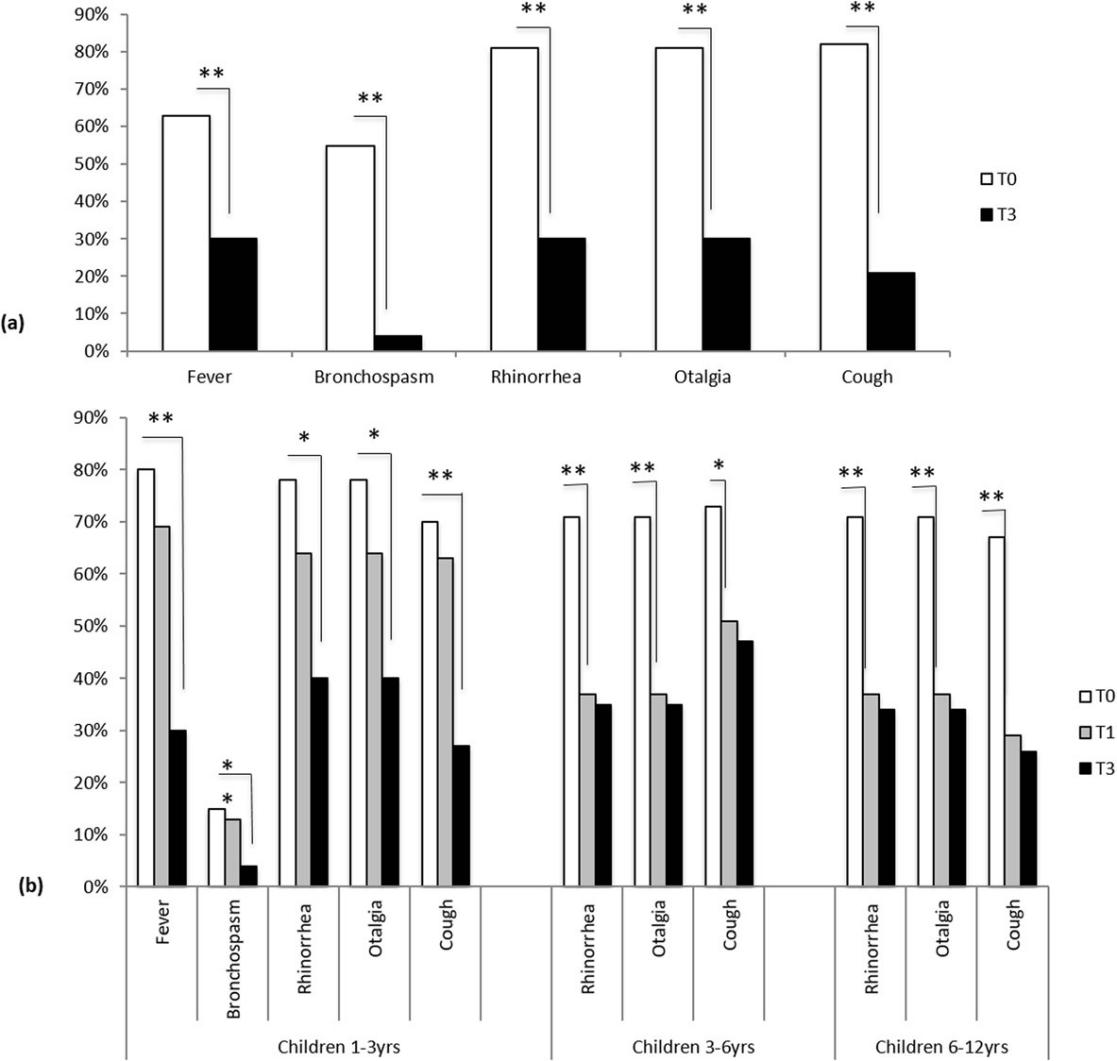
**a**, **b** Subgroup analysis*.* **a** In accordance to positive familial history for atopy, a significant decrease in number of episodes of symptoms was described (*p < 0.05). **b** According to the age, children 1–3 years old showed an improvement in all symptoms, however, they become statistically significant only at the end of the 3 months of treatment (*p < 0.05). In children 3–6 years old and 6–12 years old, the therapeutic efficacy was assessed since the first month of treatment (*p < 0.05)

In accordance to positive familial history for atopy, a significant decrease in number of episodes of symptoms was described (fever (T0: 63% vs. T3: 30% *p* < 0.001), bronchospasm (T0: 55% vs. T3: 4%; *p* < 0.001), rhinorrhea (T0: 81% vs. T3: 30%; *p* < 0.001), otalgia (T0: 81% vs. T3: 30%; *p* < 0.001), and cough (T0: 82% vs. T3: 21%; *p* < 0.001)) (Fig. 2a).

The intergroup analysis showed a significant decrease in the number of episodes of cough (chi-square: 4.34; *p* = 0.04; 95% CI: 0.80 (0.64–1.00)), bronchospasm (chi-square: 6.67; *p* = 0.01; 95% CI: 0.74 (0.59–0.94)), rhinorrea and otalgia (chi-square: 4.06; *p* = 0.04; 95% CI: 0.79 (0.62–1.00)) also in atopic children.

Conducting a subgroup analysis according to the age, it has been reported that 1–3 year-old children showed an improvement in all symptoms, however, they become statistically significant only at the end of the 3 months of treatment (fever (T0: 80% vs. T3: 30% *p* < 0.001), bronchospasm (T0: 15% vs. T3: 4%; *p* < 0.001), rhinorrhea (T0: 78% vs. T3: 40%; *p* < 0.05), otalgia (T0: 78% vs. T3: 40%; *p* < 0.05), and cough (T0: 70% vs. T3: 27%; *p* < 0.001)).

In 3–6 and 6–12 year-old children, the therapeutic efficacy in terms of rhinorrea (T0: 71% vs. T1: 37%, *p* < 0.001; T0: 71% vs. T1: 37%, *p* < 0.001, respectively for age ranges), otalgia (T0: 71% vs. T1: 37%, *p* < 0.001; T0: 71% vs. T1: 37%, *p* < 0.001), and cough (T0: 73% vs. T1: 51%, *p* < 0.05; T0: 67% vs. T1: 29%, *p* < 0.001) frequency was progressive and significant already since the first month of therapy. In both subgroups, a less percentage of episodes of fever and bronchospasm was recorded but it was not statistically significant either T1 or T3 (*p* > 0.05) (Fig. 2b).

### Safety

Although a good tolerability profile was reported, 9 children receiving *Streptococcus salivarius* 24SMBc and *Streptococcus oralis* 89a, experienced burning nose leading to interruption of therapy. However, none of the children were withdrawn from the study because of AEs.

## Discussion

Recently, increasing evidences suggested beneficial effects of probiotics in the treatment of several acute or chronic diseases [20, 21], including respiratory tract infections [15]. With this regard, studies showed that the use of probiotic strains, acting as “friendly bacteria”, in RRIs, offered great benefits for the host [15, 22]. As regards to potential pathogens, *Streptococcus salivarius* and *Streptococcus oralis* species have been proven to be capable to promote the recolonization process and to re-establish microbial balance as well as to decrease the level of potential pathogens, therefore, reducing and preventing RRIs [18, 23]. These findings were successively confirmed by the evidence that close correlations between the reduction of potential pathogens, the presence of commensal streptococci, and a lower incidence of RRIs were occurring among patients receiving probiotics treatment [18, 23].

However, despite to these encouraging findings, the clinical evidence on the role of the “bacteriotherapy” approach on RRIs treatment is still limited. Thus, in order to fill this gap, we conducted a preliminary, prospective, single-open, multi-centre study to determine the efficacy and the safety of *Streptococcus salivarius* 24SMBc and *Streptococcus oralis* 89a in the treatment of URTIs in children.

Although the observation period was short, our study revealed that *Streptococcus salivarius* 24SMBc and *Streptococcus oralis* 89a administration was significantly effective in reducing the frequency of the episodes of fever, cough, bronchospasm, rhinorrhea and otalgia in a pediatric population affected by RIs. In fact, after treatment with probiotics, a clinical improvement was reported for all symptoms. Also, the benefits of treatment were noted not only at the starting treatment but they were also maintaining themselves during treatment period. Moreover, the *Streptococcus salivarius* 24SMBc and *Streptococcus oralis* 89a administration resulted efficacy also in high-risk group such as atopics and children exposed to environmental tobacco.

Thus, it was reasonable to hypothesize that probiotics may impart health benefits to the host when administered in adequate amount of time. To date, no unanimous data are available on the probiotics supplementation timing, however, [22], taking into account the available data on safety profile of probiotics [22] and in light of our findings – no significant AEs were herein reported-, we strongly believe that a dosage regimen lasting at least 3 months it should be advised.

Also, the lack of a follow-up period in our study did not allow us to estimate the drug efficacy after treatment interruption. Thus, it is not possible to exclude that a dosage regime major than 3 months and/or a multiple cycles of therapy can represent a more adequate therapeutic approach to decrease the incidence of RIs in children. Moreover, this concept acquisition has great importance especially in younger children which, due to the impaired efficiency both of the innate and adaptive immune system, become more vulnerable to infections [24]. Our findings revealed, in fact, that patients 1–3 years old showed a progressive improvement in clinical course already after 0 month but it appeared significant at the end of the treatment, conversely, in babies older than 3 years, the therapeutic efficacy was progressive and significant since the first month of therapy. Therefore, we hypothesized that a protracted dosing schedule could better protect younger children from recurrent RIs. It remains to speculate if in addition to their effective therapeutic effects, probiotics could be also adopted as preventive treatment on RIs in this high risk group [22, 25].

On this regard, in fact, the prevention and therapeutic effectiveness of probiotics, despite to controversial results, has been also investigated in children affected by allergic diseases, population well known to experience more numerous and more severe RIs than healthy subjects [26, 27]. In order to assess the evidence that *Streptococcus salivarius* 24SMBc and *Streptococcus oralis* 89a may significantly influence also the atopy-RIs link, herein, subgroup analyses, according to familial history of atopy and atopic *status*, were conducted in enrolled population. Our study revealed that the probiotics administration resulted also effective in reducing the number of episodes of RIs both in children with parental history of atopy or atopic diseases, highlighting as probiotic consumption can be a feasible way to decrease the incidence of RIs especially in high risk group or atopic children.

However, whether on one hand our results confirmed the therapeutic efficacy of probiotics in allergic population also suffering of RIs, on the other hand, due to the lack of a follow-up period, no data can be provided on the preventive efficacy of the *Streptococcus salivarius* 24SMBc and *Streptococcus oralis* 89a in the same population.

Unfortunately, the lack of a follow-up period is not the only limit of our study. This clinical trial is an open study, without a placebo group and, it is based only on clinical outcomes without cultural investigations. However, the study was performed in a cohort of patients and in a real-life setting, thus, our results could be reasonably considered reliable data. Future studies will be needed to assess the suitable data on preventive efficacy of the investigated probiotic.

## Conclusions

Our findings suggest that Streptococcus salivarius 24SMBc and Streptococcus oralis 89a treatment is safe and seems to be effective on short-term in the treatment of RRIs. Studies involving a longer observation period are necessary to establish the real efficacy of the product for the treatment of pediatric patients affected by RRIs.

## Data Availability

The datasets used and/or analysed during the current study are available from the corresponding author on reasonable request.

## Abbreviations

AEs: Adverse events
CFU: Colony-forming units
LRTIs: Lower respiratory tract infections
RRIs: Recurrent respiratory infections
SPT: Skin Prick Test
URTIs: Upper respiratory tract infections
